# How the use of FDG PET is improving the diagnosis of dementia in a reference center in Recife, Brazil

**DOI:** 10.1055/s-0045-1808086

**Published:** 2025-05-21

**Authors:** Luisa Couceiro de Albuquerque Macêdo, Raphaelly Ribeiro Campos, Luiz Eduardo Duarte Borges Nunes, Mariana Gonçalves Maciel Pinheiro, Alberto Henrique Torres Trindade da Silva, Maria Regina Vendas Carneiro Leão, Aldson dos Santos Silva, Felipe Alves Mourato, Simone Cristina Soares Brandão, Breno José Alencar Pires Barbosa

**Affiliations:** 1 Universidade Federal de Pernambuco, Empresa Brasileira de Serviços Hospitalares, Hospital das Clínicas, Serviço de Neurologia, Recife PE, Brazil.; 2 Universidade Federal de Pernambuco, Centro de Ciências Médicas, Área Acadêmica de Neuropsiquiatria, Recife PE, Brazil.; 3 Universidade de Pernambuco, Faculdade de Ciências Médicas, Recife PE, Brazil.; 4 Universidade Federal de Pernambuco, Empresa Brasileira de Serviços Hospitalares, Hospital das Clínicas, Serviço de Medicina Nuclear, Recife PE, Brazil.; 5 Universidade Federal de Pernambuco, Centro de Ciências Médicas, Área Acadêmica de Medicina Clínica, Recife PE, Brazil.

**Keywords:** Positron-Emission Tomography, Dementia, Alzheimer Disease

## Abstract

**Background:**

Since the advent of 18F-2-fluoro-2-deoxy-D-glucose ([18F]FDG, henceforth, FDG) in the 1970s as a neurochemical tracer, FDG positron emission tomography (PET) has been used for research in dementia and to help diagnose dementing neurodegenerative disorders. However, FDG PET is still unavailable in most centers, especially those in low- and middle-income countries, and there is limited data on biomarkers from patients in diverse populations, such Latin Americans.

**Objective:**

To analyze the main indications and how the use of FDG PET helped improve the diagnosis of dementia in a specialized center in Recife, one of the largest cities in Northeastern Brazil.

**Methods:**

We retrospectively analyzed data from 62 individuals under follow-up at our center between 2018 and 2023 who had a clinical diagnosis of dementia or mild cognitive impairment and underwent FDG PET scans.

**Results:**

In 21/29 (72.4%) patients, FDG PET helped investigate the types of atypical neurodegenerative dementias; in 14/24 (58.3%), it clarified the clinical question in the investigation of early-onset dementia syndromes; and, in 9 cases, it was performed to differentiate between degenerative and non-degenerative dementias.

**Conclusion:**

These numbers may set the foundation for further longitudinal analyses and collaborative studies including participants from Northeastern Brazil.

## INTRODUCTION

Given the aging and population growth trends projected by the Global Burden of Diseases, Injuries, and Risk Factors Study (GBD) 2019, 1 the global number of people with dementia is expected to increase from 57.4 million cases in 2019 to 152.8 million cases in 2050. Although to date effective treatments for neurodegenerative diseases are limited, it is critical to establish an accurate diagnosis so that patients can be screened for appropriate care and management. 2

The clinical diagnosis of patients with dementia can be challenging, as different types of neurodegenerative diseases exhibit overlapping symptoms in the clinical presentation. This happens especially in cases of early onset of the disease (age < 65 years), in atypical forms of Alzheimer's disease (AD; such as frontal or language variants), or in cases in which the clinical symptoms do not fulfill the criteria for a specific syndrome. 3

Since the advent of 18F-2-fluoro-2-deoxy-D-glucose ([18F]FDG, henceforth, FDG) in the 1970s as a neurochemical tracer, FDG positron emission tomography (PET) has been used for research in dementia and clinical applications. 4 The FDG imaging biomarker measures brain metabolic activity, and the extent of metabolic impairment is assessed through FDG PET to indicate synaptic damage to diagnose neurodegenerative disorders. In the brain areas first affected by the disease, FDG PET can find decreased activity. The pattern of reduced metabolism is also used to help diagnose different neurodegenerative disorders based on what we currently know about the brain circuits involved, making it possible to differentiate central dementia disorders, including AD, frontotemporal dementia (FTD), and Lewy body dementia (LBD). 5 6

In a consensus article, 7 experts in nuclear medicine and neurology recommended using FDG PET alongside clinical evaluations for neurodegenerative diseases; they explicitly advised its use to aid in the diagnosis of atypical AD and to differentiate it from LBD, FTD, and vascular cognitive impairment (VCI). The Brazilian Academy of Neurology (Academia Brasileira de Neurologia, ABN, in Portuguese) recently recommended 8 FDG PET as a neurodegeneration biomarker to evaluate language- and behavioral-predominant dementias, as well as corticobasal syndrome. Patterns of hypometabolism suggestive of underlying AD pathology could help clinicians select more appropriate candidates to test cerebrospinal fluid biomarkers. This aligns with the current amyloid/tau/neurodegeneration (A/T/N) framework for the biomarker-based diagnosis of AD, with FDG PET listed as a neurodegeneration biomarker. 9

18F-2-fluoro-2-deoxy-D-glucose PET is still unavailable in most centers, especially in low—and middle-income countries; 10 moreover, there is limited data on biomarkers from patients in diverse populations, such those from Latin America, where FDG PET could pose as a reasonable substitute for tau PET in locations where it is unavailable due to its good negative correlation with areas of tracer hyperuptake for phosphorylated tau (p-tau). 8 11 Another challenge is developing protocols that involve appropriate patient selection by dementia specialists and precise diagnosis by nuclear medicine specialists with the routine use of semiautomated quantification to assist visual analyses. 7

In the current study, we aimed to report our experience throughout the first 5 years of routine FDG PET use by describing the main indications and results of this method at a specialized center in Recife, one of the largest cities of Northeastern Brazil, a region with social disparities that has been considered underrepresented in dementia cohorts. 12 Additionally, we illustrated the use of FDG PET based on two cases.

## METHODS

### Study design

The present study is a descriptive, health records-based case series with clinical and imaging characterization of dementia patients followed up at the Memory Clinic of the Neurology Division at Hospital das Clínicas, Universidade Federal de Pernambuco, in Recife. The study was approved by the institutional Ethics Committee under protocol CAAE 24235419.2.0000.8807.

### Population

We retrospectively analyzed all individuals under follow-up at our center between 2018 and 2023 who had a clinical diagnosis of dementia or of mild cognitive impairment and underwent FDG PET scans. Cognitive and behavioral neurologists routinely evaluated all patients and classified them according to the guidelines proposed by the ABN. 13 14 15 16 17 We excluded patients whose initial clinical impressions were of conditions other than dementia and mild cognitive impairment, or those who did not undergo PET.

### Imaging protocols and diagnostic patterns

Before the examination, the patient should fast for 4 to 6 hours, refraining from all food and drinks except water, to decrease glucose and insulin levels. Glucose solution and parenteral nutrition should also be stopped 4 to 6 hours before the exam. The patient should be encouraged to stay hydrated by drinking water. During the intravenous injection of FDG in the Nuclear Medicine Service, the patient should rest for 30 to 45 minutes to minimize muscle uptake. Throughout this resting period, the patient should be kept warm and shielded from auditory and visual stimuli while keeping their eyes open for the tracer uptake phase.

The radiopharmaceutical used for brain studies is FDG with a dose of 5 millicuries (mCi). The images are acquired 30 to 45 minutes after the tracer injection. Maintaining consistent acquisition time across all exams is essential to accurately compare uptake intensity and the standardized uptake value (SUV). Patients should empty their bladder and remove metallic objects from their clothing before the images are acquired. The PET/computed tomography (CT) scanner used for imaging is the Discovery 710 model from General Electric, with 128 channels. During the acquisition, the patient must keep their head still and lie on their back with their arms down. Images of only the patient's skull will be obtained for 10 minutes.

### Initial diagnosis and FDG PET assessment

**Figure 1:**
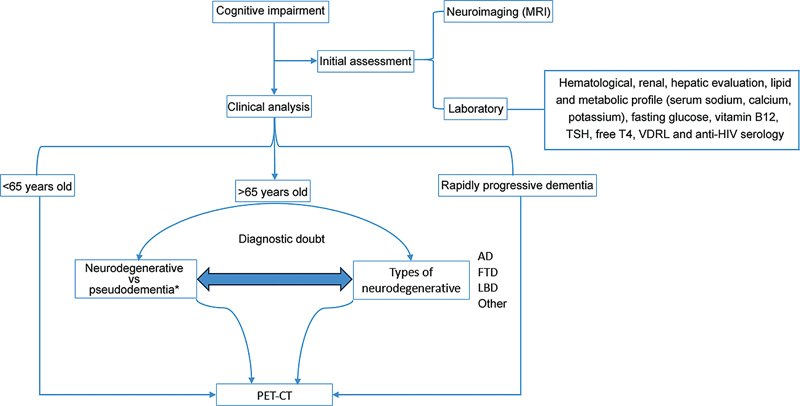
Patients with cognitive decline and a syndromic diagnosis of dementia or mild cognitive impairment underwent 18F-2-fluoro-2-deoxy-D-glucose positron emission tomography (FDG PET) whenever there was diagnostic doubt regarding different dementias, degenerative versus non-degenerative causes, or dementias overlapping with other diseases.

Patients with cognitive decline and a syndromic diagnosis of dementia or mild cognitive impairment underwent FDG PET whenever there was diagnostic doubt regarding different dementias, degenerative versus non-degenerative causes, or dementias overlapping with other diseases ( Figure 1 ). It is worth noting that we did not perform FDG PET for patients who had typical AD, a probable clinical diagnosis of other dementia syndromes such as FTD, LBD or VCI, or an evident psychiatric disorder as the cause of cognitive decline, since there is no clinical indication for the exam in these cases. Nuclear medicine specialists performed a visual analysis followed by a quantitative analysis with CortexID Suite software and established the diagnosis according to standardized patterns. 18 19

### Variables and outcomes

The variables included in the present study were the patients' demographics, scenarios for indication of the scan, and FDG PET diagnostic impression. As shown in Table 1 , most scans were performed in the following scenarios: degenerative versus non-degenerative dementias; differential diagnosis regarding types of neurodegenerative dementias; and investigation of early-onset or atypical dementia syndromes. The diagnoses of the FDG PET consisted of AD, LBD, and FTD—including progressive supranuclear palsy (PSP) or corticobasal degeneration (CBD) and VCI following current established patterns. 6 19 The exam was considered diagnostic if it could answer the initial clinical question, and not diagnostic if the scan showed non-specific changes in brain metabolism or if it exhibited other responses unrelated to the initial diagnostic question (example: FDG PET suggested a diagnosis of VCI in the absence of vascular lesions in the structural imaging). It is noteworthy that it was not our objective to perform sensitivity or specificity analyses, since the patients did not have access higher-accuracy methods such as biomarkers or postmortem analysis at our center.

**Table 1:** Clinical and Imaging characterization of the study sample

Patients' characteristics (N = 62)	
Mean age in years (range)	71.09 (39–95)
Women: n (%)	36 (58.1%)
FDG PET diagnosis: n (%)	
*AD*	19 (30.64%)
*LBD*	8 (12.9%)
*FTD*	8 (12.9%)
*NSC*	14 (22.6%)
*NC*	11 (17.74%)
Scenarios for FDG PET: n (%)	
*Types of neurodegenerative dementias*	29 (46.77%)
* AD versus FTD*	8 (12.9%)
* AD versus LBD*	4 (6.45%)
*Early-onset dementias*	24 (38.77%)
*Degenerative versus non-degenerative dementias*	9 (14.51%)

Abbreviations: AD, Alzheimer's disease; FDG PET, 18F-2-fluoro-2-deoxy-D-glucose positron emission tomography; FTD, frontotemporal dementia; LBD, Lewy body dementia; NSC, non-specific changes in metabolic view; NC, no changes.

## RESULTS

**Figure 2:**
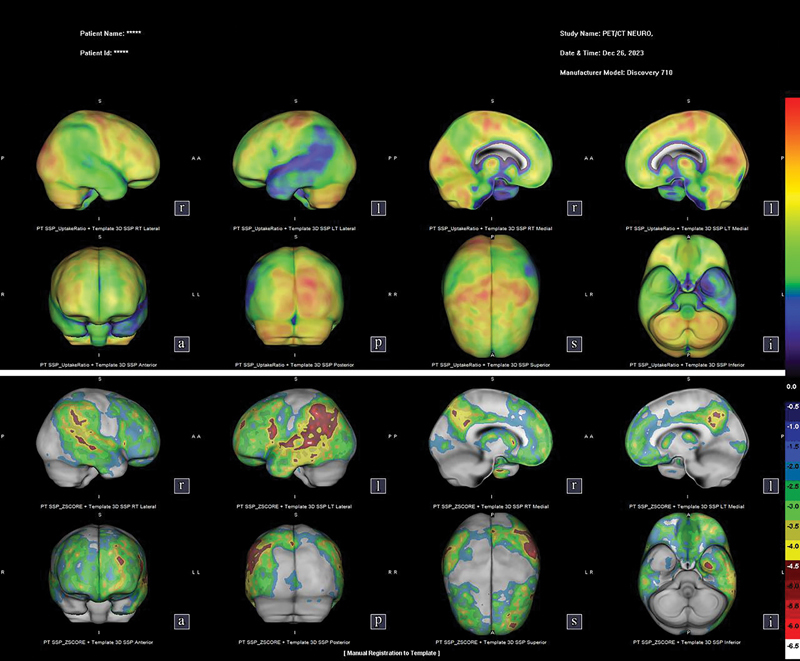
Case 1 - FDG PET scan showing glycolytic hypometabolism in the bilateral temporoparietal cortex, more pronounced on the left, suggestive of Alzheimer's disease.

As shown in Table 1 , 62 patients underwent FDG PET in the investigations of cognitive syndromes. Their mean age was of 71.09 (range: 39–95) years, and 58% were female. While 15 scans were non-diagnostic and showed non-specific changes, 19 suggested AD, 8 suggested LBD, 11 suggested FTD, and 9 showed no changes. In 21/29 (72.4%) patients, the scan helped investigate types of atypical neurodegenerative dementias; in 14/24 (58.3%), it clarified the clinical question in the investigation of early-onset dementia syndromes (as illustrated by case 1 in Box 1 , Figure 2 ); and, in 9 cases, the exam was performed to differentiate between degenerative and non-degenerative dementias ( Table 2 ).

**Box 1:** Illustrative case report 1

Case 1: A 56-year-old female patient with higher education and right-hand dominance. The condition began approximately 2 years before the first assessment, with difficulty finding words and communication impairments. She also had difficulty remembering dates and events of the day. During the interview, she was alert and showed appropriate behavior, but in the language assessment there was a severe loss of fluency, with pauses and hesitations. There was marked anomia and frequent use of generic terms (e.g. “thing, that”). Sentence repetition was also markedly impaired, but comprehension was relatively spared, as she could understand simple questions and commands. In the Mini-Mental State Examination, she scored 18/30 and, on the Pfeffer Questionnaire, 20/30. She named 5 figures from Addenbrooke's Cognitive Examination and recognized them all after clues. Upon neurological examination, she did not have parkinsonism, and other impairments were absent. Magnetic resonance imaging of the skull showed signs of brain volumetric reduction with predominance in the high parietal convexities, notably on the left, as well as slight asymmetry of the Sylvian fissures, wider on the left. As this was an atypical dementia syndrome of early onset, an 18F-2-fluoro-2-deoxy-D-glucose positron emission tomography scan was requested, which revealed glycolytic hypometabolism in the bilateral temporoparietal cortex, more pronounced on the left. The study was suggestive of Alzheimer's disease.

**Table 2:** Comparative table of the indication of FDG PET and its percentage of examinations that assisted in the initial clinical question

	**AD**	**LBD**	**FTD**	**NSC**	**NC**	** Diagnostic scans ^a^ /total scans (%) **
Differential diagnosis of neurodegenerative dementias	10	5	6	4	4	21/29 (72.4%)
AD versus FTD ^b^	4	0	0	2	2	4/8 (50%)
AD versus LBD ^b^	2	1	1	0	0	3/4 (75%)
Investigation of early-onset dementia syndromes	7	3	4	6	4	14/24 (58.3%)
Degenerative versus non-degenerative dementias	2	0	0	4	3	5/9 (55.5%)
Total	19	8	10	14	11	40/62 (64.5%)

Abbreviations: AD, Alzheimer's disease; FDG PET, 18F-2-fluoro-2-deoxy-D-glucose positron emission tomography; FTD, frontotemporal dementia; LBD, Lewy body dementia; NSC, non-specific changes in metabolic view; NC, no changes.

Notes: ^a^ The numbers represent the frequency of diagnostic scans/total scans in each scenario. The exam was considered diagnostic if it could answer the initial clinical question. It was considered non-diagnostic if the scan showed non-specific changes in brain metabolism or if it exhibited other responses unrelated to the initial diagnostic question. ^b^ Subgroups in the scenario of differential diagnosis of neurodegenerative dementia.

In evaluating different neurodegenerative dementias, FDG PET helped answer the clinical question in 21/29 (72.4%) patients. It is noteworthy that one scenario in which FDG PET did not help much was in diagnosing AD versus FTD: the initial question between these two diseases was only answered in 50% of the cases, supporting one condition or another by showing one typical pattern (that is, posterior hypometabolism in AD as opposed to frontotemporal impairment in FTD). On the other hand, when considering the differential diagnosis of AD and LBD, the results were helpful in 75% of the cases (as illustrated by case 2 in Table 4 ). Table 2 compares the initial clinical question with the FDG PET results.

## DISCUSSION

The present study described the main indications for FDG PET and how the results answered the clinical question. The exam was mainly used to clarify atypical degenerative causes (29 cases), to evaluate early-onset or atypical dementia syndromes (24 cases), and in the differential diagnosis of degenerative and non-degenerative causes of dementia, such as pseudodementia due to psychiatric conditions (9 cases). The exam proved most helpful in the first and second scenarios, in which it improved diagnosis in 72% and 58.3% of all cases respectively.

**Figure 3:**
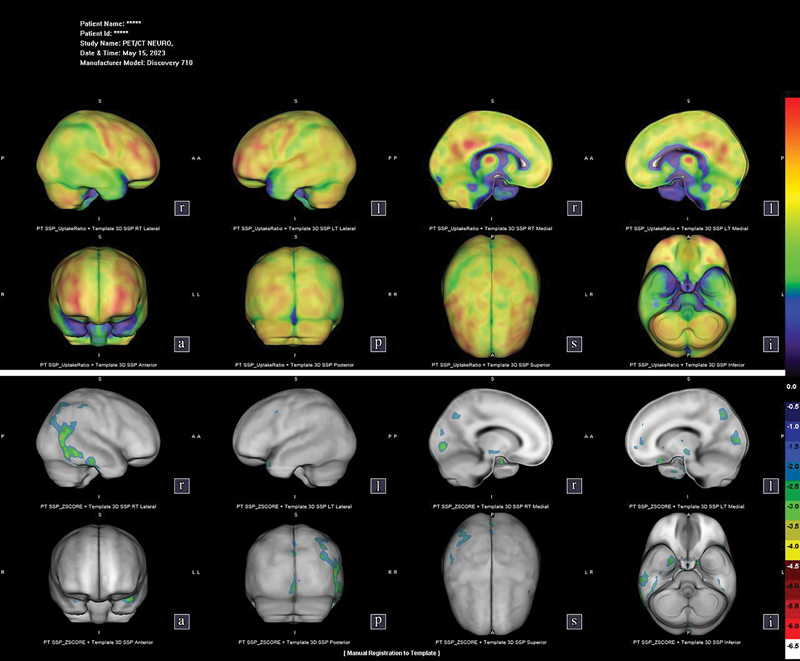
Case 2 - FGT PET scan showing asymmetry in glycolytic metabolism between the cerebral hemispheres. There is slight hypometabolism in the temporoparieto-occipital regions, which is more evident on the right side. It is important to note that the metabolism in the posterior cingulate is relatively preserved, a pattern suggestive of the cingulate island sign.

Positron-emission tomography is well-established in dementia centers that handle challenging, atypical cases, such as those reported in Boxes 1 and 2 , Figures 2 and 3 . In these situations, the standard neurological evaluation through magnetic resonance imaging often proves insufficient, and molecular imaging becomes essential in identifying the etiological diagnosis through different patterns of brain metabolism. However, the diagnostic accuracy of PET is variable, depending on precise clinical indications, which require dementia specialists, in addition to the learning curve of the nuclear medicine sector. Furthermore, FDG PET is limited to a few centers in Brazil, usually with an oncological focus, as the use of FDG is more easily reimbursable in this context.

**Box 2:** Illustrative case report 2

Case 2: A 71-year-old man, with only 5 years of incomplete primary education, who had been retired for 5 years from his job as a cashier in a bakery. He began experiencing cognitive impairment in 2022, presenting repetitive speech noted by his relatives, forgetting where he had placed everyday objects such as his wallet and cell phone, burning meals while cooking, and leaving tasks half done. Furthermore, he had difficulty remembering previously discussed facts or information broadcasted on television. When asked, he reported “restless sleep” with diurnal somnolence, with no report of visual alucinations. Six months later, he developed spatial disorientation outside the home, which was reported. During the neurological evaluation, he was alert, attentive, and cooperative, presenting mild parkinsonism, with no report of possible causing medications; the Mini-Mental State Examination reported losses in temporal orientation, recall memory, and drawing. The functional losses were moderate, with a score of 7/30 on the Pfeffer Functional Activities Questionnaire. The laboratory tests for reversible causes of dementia showed no abnormalities, and a magnetic resonance imaging scan only revealed findings of mild microangiopathy Fazekas 1. The FDG PET scan showed significant asymmetry between the cerebral hemispheres, with slight hypometabolism most pronounced in the right temporoparieto-occipital regions. Additionally, the preservation of posterior cingulate metabolism resembled the “island sign”. Based on the combination of imaging results and clinical symptoms, the patient was diagnosed with Lewy body dementia.

Although it is considered an advanced method of molecular imaging diagnosis, FDG PET is viewed as a biomarker of neurodegeneration, thus being less specific compared to other methods that confirm the presence of proteins in vivo (such as PET with amyloid ligand). Studies that report on the diagnostic accuracy of FDG PET usually lack comparisons to gold-standard methods such as post-mortem pathology. Another issue is the reliance on so-called typical patterns of hypometabolism that rely solely on the overlap between clinical syndromes and pathology. 6 In this sense, the current trend in research is using molecular neuroimaging techniques to detect cognitive decline, which have focused on identifying misfolded protein deposits. These innovative methods became feasible thanks to the development of radiotracers that bind to these pathological proteins. 19

In the current study, when evaluating different neurodegenerative dementias, FDG PET helped answer the clinical question in 21/29 (72.4%) patients. The exam probably enabled a better classification by showing specific hypometabolism patterns in cases of atypical dementia syndromes such as dementia with parkinsonism, including PSP, corticobasal syndrome (CBS), and language- or behavioral-predominant dementias. When considering the differential diagnosis of AD versus FTD in our cases, the results were only helpful in 50% of the cases. These results contradict those of the literature, 20 since most studies report that FDG PET helps differentiate typical AD patterns and discriminates AD from FTLD with more than 85% of sensitivity and specificity. Prefrontal and/or anterior temporal hypometabolism are observed in FTD, whereas, in typical AD the pattern is bilateral predominant medial and lateral temporoparietal (with less pronounced prefrontal) hypometabolism.

In most cases, the hypometabolic patterns of FTD and AD are clearly distinguished. Since frontal regions can be affected in AD and the temporoparietal cortex, in FTD, there may be a significant overlap between the syndromes. In these cases, which are generally correlated with the severity of the disease stage, the diagnosis cannot be reached based on clinico-neuropsychological evaluation, and FDG PET and amyloid biomarkers may be more informative. 21

When differentiating between LBD and AD, our results were similar to those of the literature, with a sensitivity ranging from 70 92%, specificity, from 74 to 100%, and accuracy, from 72 to 96%. Therefore, the evidence supporting the clinical usefulness of FDG PET is ranked as good. In this context, however, radiopharmaceuticals targeting the brain presynaptic dopaminergic pathway or cardiac postganglionic norepinephrine transporter are more accurate in differentiating LBD from AD. Patients with LBD have demonstrated occipital hypometabolism bilaterally, with up to 80% sensitivity and 100% specificity. 22 Moreover, increased metabolism levels were identified in the posterior cingulate compared with the sum of the precuneus and cuneus; the latter has been named the *cingulate island sign* , and it is more specific than occipital hypometabolism for LBD, 23 as illustrated in Table 4 .

Early-onset dementia patients often present with atypical clinical symptoms, hampering an accurate clinical diagnosis that may include vascular, inflammatory, and atypical degenerative syndromes such as CBS or primary progressive aphasias, as illustrated in Table 3 . Our results showed interesting rates of diagnostic scans (58.3%), which align with those of the literature. As reported in some recent studies, FDG PET could help differentiate the underlying pathology in patients with CBS. 24 25 In patients with “atypical/unclear dementia,” an FDG PET scan led to a diagnostic change in 59.5% of the cases, and it increased the prescription of cholinesterase inhibitors from 13.8 to 38.3%. 26

Suspicions of pseudodementia and other diseases progressing to cognitive impairment were also evaluated. The FDG PET was especially useful in those patients with atypical presentations and substantial psychological overlay. Our results in this subgroup were somewhat inconclusive: in 9 cases, the exam was used to differentiate degenerative or non-degenerative causes, and 3 were negative, 4 showed non-specific results, and 2 were consistent with AD. Depression and the use of medications with anticholinergic effects are common causes of potentially-reversible cognitive impairment. Reports 27 suggest that late-onset depressive symptoms or syndromes can be a prodrome of cognitive decline or early manifestations of dementia, being shared among patients with mild and moderate dementia. Subtle frontal hypometabolism may be found in some patients with severe depression, whereas a normal FDG PET scan in a demented patient offers strong evidence supporting pseudodementia. This recommendation especially applies to patients with apparent overt dementia on cognitive testing. However, when it comes to the strength of the evidence of FDG PET in the scenario of pseudodementia in general, the fundamental limitation is the paucity of evidence on which to base recommendations. 21

More studies on neuroimaging in dementia in Brazil are needed, as the country has its own social, cultural, racial, and regional peculiarities. Poor access to neuroimaging is generally considered a main limitation to diagnose dementia in Brazil, as opposed to high-income countries such as the United Kingdom. 10 In a recent ABN survey, 28 39 out of 256 physicians considered poor access to functional neuroimaging a significant challenge in the diagnostic framework of frontotemporal dementia.

Some limitations to the current study should be acknowledged. The diagnosis was not confirmed postmortem or using on protein-based biomarkers, and the final clinical diagnosis was exclusively based on a review of case notes. Moreover, the present is a purely descriptive analysis, and no statistical testing was performed to compare clinical and FDG PET diagnostic accuracies. On the other hand, it is important to note that this sample included a significant number of patients who underwent an FDG PET in a region considered underrepresented in dementia research.

In conclusion, the present study reports the first 5 years of experience using FDG PET to aid in the diagnosis of dementia at our center. As a main result, in 21/29 (72.4%) patients, the scan helped investigate types of atypical neurodegenerative dementias; in 14/24 (58.3%), it clarified the clinical question in investigating early-onset dementia syndromes; and, in 9 cases, the exam was performed to differentiate between degenerative and non-degenerative dementias. These numbers may set the foundation for further longitudinal analyses and collaborative studies including participants from Northeastern Brazil.
